# Impact of CRAMP-34 on *Pseudomonas aeruginosa* biofilms and extracellular metabolites

**DOI:** 10.3389/fcimb.2023.1295311

**Published:** 2023-12-13

**Authors:** Shiyuan Wang, Chengjun Ma, Jinying Long, Peng Cheng, Yang Zhang, Lianci Peng, Lizhi Fu, Yuandi Yu, Dengfeng Xu, Suhui Zhang, Jinjie Qiu, Yuzhang He, Hongzao Yang, Hongwei Chen

**Affiliations:** ^1^ College of Veterinary Medicine, Southwest University, Chongqing, China; ^2^ Collaborative Innovation Institute National Center of Technology Innovation for Pigs, Chongqing, China; ^3^ College of Veterinary Medicine, Nanjing Agricultural University, Nanjing, China; ^4^ Immunology Research Center, Medical Research Institute, Southwest University, Chongqing, China; ^5^ Institute of Veterinary Medicine Academy of Animal Sciences, Chongqing, China

**Keywords:** *Pseudomonas aeruginosa*, CRAMP-34, anti-biofilm peptides, extracellular metabolites, synergistic effect

## Abstract

Biofilm is a structured community of bacteria encased within a self-produced extracellular matrix. When bacteria form biofilms, they undergo a phenotypic shift that enhances their resistance to antimicrobial agents. Consequently, inducing the transition of biofilm bacteria to the planktonic state may offer a viable approach for addressing infections associated with biofilms. Our previous study has shown that the mouse antimicrobial peptide CRAMP-34 can disperse *Pseudomonas aeruginosa* (*P. aeruginosa*) biofilm, and the potential mechanism of CRAMP-34 eradicate *P. aeruginosa* biofilms was also investigated by combined omics. However, changes in bacterial extracellular metabolism have not been identified. To further explore the mechanism by which CRAMP-34 disperses biofilm, this study analyzed its effects on the extracellular metabolites of biofilm cells via metabolomics. The results demonstrated that a total of 258 significantly different metabolites were detected in the untargeted metabolomics, of which 73 were downregulated and 185 were upregulated. Pathway enrichment analysis of differential metabolites revealed that metabolic pathways are mainly related to the biosynthesis and metabolism of amino acids, and it also suggested that CRAMP-34 may alter the sensitivity of biofilm bacteria to antibiotics. Subsequently, it was confirmed that the combination of CRAMP-34 with vancomycin and colistin had a synergistic effect on dispersed cells. These results, along with our previous findings, suggest that CRAMP-34 may promote the transition of PAO1 bacteria from the biofilm state to the planktonic state by upregulating the extracellular glutamate and succinate metabolism and eventually leading to the dispersal of biofilm. In addition, increased extracellular metabolites of myoinositol, palmitic acid and oleic acid may enhance the susceptibility of the dispersed bacteria to the antibiotics colistin and vancomycin. CRAMP-34 also delayed the development of bacterial resistance to colistin and ciprofloxacin. These results suggest the promising development of CRAMP-34 in combination with antibiotics as a potential candidate to provide a novel therapeutic approach for the prevention and treatment of biofilm-associated infections.

## 1 Introduction

Bacterial biofilms consist of aggregate cells that adhere to various surfaces, enveloped within a self-produced extracellular polymeric substance (EPS) matrix (Afrasiabi et al., 2021). This composition is crucial for bacterial survival. Within biofilms, bacteria demonstrate resistance to stress conditions and evade host immune responses (Petrova and Sauer, 2016). Notably, biofilms are linked to chronic infections and display resistance to antimicrobial treatments, being up to 1,000 times more resilient than their planktonic counterparts. This disparity underscores the daunting challenge of developing effective treatments against biofilm formation (van Wolferen et al., 2018). While earlier antibiofilm research primarily targeted inhibiting biofilm formation, recent studies have delved into the mechanisms of bacterial dispersion from biofilms. Thus, bacterial dispersion emerges as a prospective target for antibiofilm drug development. The transition of biofilm bacteria to a planktonic state renders them more susceptible to antimicrobial agents and immune attacks. Consequently, leveraging bacterial dispersion is gaining traction as a promising strategy for biofilm management (Rumbaugh and Sauer, 2020).

*Pseudomonas aeruginosa* (*P. aeruginosa*) is a significant pathogen responsible for both acute and chronic infections in humans and animals. It results in diverse conditions, including cystic fibrosis pneumonia, Pressure sore infection, Burn wound infection in humans, otitis in livestock and pets, and hemorrhagic pneumonia in foxes (Skariyachan et al., 2018; Malhotra et al., 2019; de Sousa et al., 2021; Rossi et al., 2021). Additionally, *P. aeruginosa* is renowned for its biofilm formation capabilities and serves as a model organism for biofilm research (Azam and Khan, 2019). Various strategies have been employed to counteract *P. aeruginosa* biofilms, including quorum sensing regulators, bioactive molecules, bacteriophages, antimicrobial peptides (AMPs), and plant extracts (Afrasiabi et al., 2021; Mehdizadeh et al., 2021; Saeloh and Visutthi, 2021). Given the rising antibiotic resistance, AMPs are increasingly viewed as potential antibiotic alternatives (Luo and Song, 2021). Presently, several AMPs have demonstrated efficacy in inhibiting biofilm formation and eliminating established biofilms (Pletzer et al., 2016; Mishra et al., 2017; Pulido et al., 2018; Dostert et al., 2019; Moussa and Aparicio, 2020). However, research on biofilm dispersion induced by AMPs is still limited.

In a previous study, we demonstrated that a mouse antimicrobial peptide CRAMP-34 (a cathelin-related antimicrobial peptide) could disperse *P. aeruginosa* biofilms. We also explored the potential mechanism behind CRAMP-34’s ability to eradicate these biofilms using multi-omics approaches (Zhang et al., 2022). Understanding the changes in extracellular metabolites in biofilm bacteria is pivotal for comprehensively elucidating CRAMP-34’s mechanism of biofilm dispersion. In this research, we scrutinized the extracellular metabolic profiles of *P. aeruginosa* biofilms post CRAMP-34 treatment to delve deeper into its biofilm-dispersing mechanism. Additionally, we evaluated the synergistic impact of combining CRAMP-34 with antibiotics against dispersed bacteria. Our results substantiate the biofilm-dispersing effect of CRAMP-34, marked by significant shifts in some extracellular metabolites such as glutamate, succinate, myoinositol, palmitic acid, and oleic acid. Moreover, combining CRAMP-34 with an antibiotic exhibited enhanced efficacy against dispersed bacteria and concurrently delayed the development of antibiotic resistance. These insights underscore the potential of leveraging CRAMP-34 as a biofilm dispersant in tandem with antibiotics, offering an innovative therapeutic strategy for mitigating biofilm associated infections.

## 2 Materials and methods

### 2.1 Bacterial strain and growth conditions

*Pseudomonas aeruginosa* PAO1 used in this study was purchased from China General Microbiological Culture Collection Center (CGMCC). PAO1 was grown in lysogeny broth (LB) medium (per liter, 5g of yeast extract, 10g of tryptone, 10g of NaCl [pH 7.2 to 7.4]). LB nutrient agar, Luria-Bertani broth and Mueller-Hinton broth were purchased from Qingdao Haibo Biotechnology Co., Ltd.

### 2.2 Peptide and antibiotics

CRAMP-34 (GLLRKGGEKIGEKLKKIGQKIKNFFQKLVPQPEQ) was synthesized by Suzhou Qiangyao Biotechnology Company, and obtained at a purity grade of > 90% by HPLC. Antibiotics were purchased from Meilun Bio-Technology Co., Ltd. and Shanghai Yuanye Bio-Technology Co., Ltd., including Meropenem (MER, lot number D0715A),Imipenem (IMP, lot number A9418A), Piperacillin (PIP, lot number D1109A), Ceftazidime (CAZ, lot number J123A), Cefquinome Sulfate, (CQN, lot number 111633), Cefoperazone Acid (CFP, lot number A0320A), Amikacin Sulfate (AMK, lot number A03237A), Azithromycin (AZM, lot number F1210A), Roxithromycin (ROX, lot number M0309A), Vancomycin (VAN, lot number O0809A), Gentamicin Sulfate (GEN, lot number S22J11Y116233), Enrofloxacin (ENX, lot number DK03-1802065) and Colistin Sulfate (COL, lot number S11N10Y102709).

### 2.3 Establishment and intervention of biofilm *in vitro*

Mature biofilms were prepared as previously described with minor modifications (Zhang et al., 2022). Briefly, biofilms were preformed by adding the test bacterial solution into 96-well plates (Corning^®^3599; Corning Inc., Corning, NY, USA) and incubating at 37°C for 72 h. Afterwards, the plates were washed three times with phosphate-buffered saline (PBS). Next, 100 µL of each concentration of CRAMP-34 was added to the plate and incubated at 37°C for 1 h. In order to meet the requirements of omics sample detection, the biofilm samples were still prepared in cell culture flasks (25 cm^2^; Corning^®^430168; Corning Inc.).

### 2.4 Determination of biofilm biomass

The biomass of biofilms was assessed using the crystal violet staining method as described previously (Zhang et al., 2022). The supernatant of biofilm samples after CRAMP intervention was discarded, and gently wash the biofilm twice with sterile PBS. The biofilm was fixed with 99% methanol for 10 minutes, followed by air drying, staining with 0.04% crystal violet solution for 20 min, and washing with sterile PBS. Then, 33% acetic acid was used to dissolve the bound crystal violet, and absorbance was measured at *OD*_600_ nm.

### 2.5 Metabolic viability assay of biofilm bacteria

The metabolic viability of biofilm bacteria was analyzed by the formazan dye-based MTT (3-(4,5-dimethylthiazol-2-yl)-2,5-diphenyltetrazolium bromide) assay (Malhotra et al., 2019). As previously mentioned, biofilm samples after CRAMP-34 intervention were gently washed twice, and then 100 μL of 0.05% (w/v) MTT solution was added to each well. Plates were incubated at 37°C in the dark for 3 h. After incubation, the formazan crystals were dissolved with 100 μL of DMSO. Absorbance was measured at *OD*_520_ nm.

### 2.6 Confocal laser scanning microscopy (CLSM)

To further confirm the effect of CRAMP-34 on dispersing PAO1 biofilms, the morphology of biofilms was observed by CLSM as described previously with some modifications (Zhang et al., 2022). Five hundred μL of the bacterial solution (*OD*_600 _= 0.1) was added to an 8-well chambered cover glass (1.5 Borosilicate glass, Lab-Tek II chambered coverglass, Rochester, NY, USA), and the medium was replaced with fresh medium every 24 h. After incubation for 3 days at 37°C, the biofilm was treated with CRAMP-34 (62.5 μg/mL) at 37°C for 1 h. After the incubation, the biofilm was washed with 0.9% (w/v) NaCl and stained for 20 min in the dark at room temperature using a Filmtracer™ LIVE/DEAD™ Biofilm Viability kit (Cat. No., L10316; Molecular Probes, Thermo Fisher Scientific, Waltham, MA, USA) (final concentrations: 5 μM SYTO9 and 30 μM propidium iodide (PI)). The biofilm was imaged with a confocal laser scanning microscopy (ECLIPSE Ti2; NIKON, Tokyo, Japan). Signals were recorded using the green (SYTO9, excitation of 488 nm) and red (PI, excitation of 561 nm) channels. The three-dimensional (3D) image was constructed by stacking multiple images with different Z values (z-stack). Three fields of view were randomly selected for each well, and the test was repeated at least three times independently. The total fluorescence intensity of live/dead bacteria, the number, volume and the bottom area of biofilms were analyzed by BiofilmQ software.

### 2.7 Metabolomics analysis

Biofilm samples were prepared as described above. Preformed biofilms were treated by CRAMP-34 at 62.5 μg/mL for 1 hour, then the upper culture medium in cell culture flasks was collected and centrifuged at 3000 rpm for 10min at 4°C, and 800 μL of supernatant was collected and frozen in liquid nitrogen for storage. There were 6 samples in both the CRAMP-34 group and the control group. The samples were sent to Beijing Oweisen Gene Technology for metabolomics detection and data analysis. The workflow was as previously reported (Zhang et al., 2022): the samples were analyzed by liquid chromatography mass spectrometry (LC-MS). The LC-MS data were extracted and preprocessed using MasterView (SCIEX). The resulted three-dimensional data involving the peak number, sample name, and normalized peak area were fed to R package metaX. For principal component analysis (PCA) and orthogonal partial least square-discriminate analysis (OPLS-DA). PCA showed the distribution of origin data. There were six replicates in each group and five replicates of non-experimental standard samples were also tested. The differentially expressed metabolites were identified based on both the variable importance in the projection (VIP) value (> 1) and the *p*-value (< 0.05).

### 2.8 Combination therapy against dispersed bacteria

The modified time killing curve (TKC) method was used to determine the dynamic bactericidal activities of CRAMP-34 in combination with antibiotics against dispersed bacteria, and TKCs were constructed by plotting mean colony counts (log10 CFU/mL) *vs* time (Dosler and Karaaslan, 2014). The method of obtaining dispersed bacteria was described previously (Zhang et al., 2022). Synergy or antagonism is defined as the combination of drugs can reduce or increase the number of biofilm bacteria more than 2 log_10_ (CFU/mL) when used alone, if the range of change is within 2 log_10_ (CFU/m L), it is defined as additive action (Dosler and Karaaslan, 2014). According to the results of pretest, the concentration of CRAMP-34 was fixed as 1/4 MIC, and then combined with the sub inhibitory concentration of different antibiotics (1/2, 1/4, and 1/8 MIC).

### 2.9 Inducible resistance

Bacterial resistance was induced *in vitro* using a modified multi-step method (Zhu et al., 2017). The method of obtaining dispersed bacteria was described previously (Zhang et al., 2022). Planktonic bacteria and biofilm dispersion bacteria were induced at the initial 1/4 MIC concentration of antimicrobial agents every 24 hours. The latest MIC was measured every 2 generations and the drug concentration was changed according to the latest results. If the bacteria did not grow well or did not grow, the drug concentration would be reduced or the culture time would be prolonged.

### 2.10 Statistical analyses

Data were analyzed by GraphPad Prism 8.0 software. Student’s t-tests were used to calculate the statistical signifcance. Signifcant diferences are indicated as *P < 0.05,**P < 0.01 and ***P < 0.001.

## 3 Results

### 3.1 Assay to biofilm biomass and metabolic activity of biofilm bacteria

**Figure 1:**
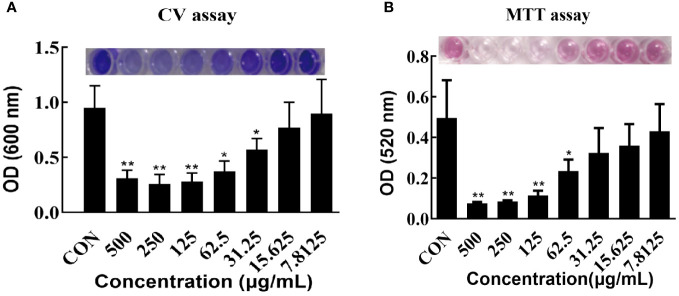
Effect of CRAMP-34 on PAO1 pre-biofilm biomass and metabolic viability. **(A)** PAO1 biofilms were cultured for 72 hours at 37°C in 96-well plates. these biofilms were exposed to CRAMP-34 for 1 hour. Biomass quantification was conducted using crystal violet (CV). **(B)** After culturing PAO1 biofilms under similar conditions and post-exposure to CRAMP-34, the metabolic viability of the biofilm-associated bacteria was assessed using the formazan-based MTT assay. The significance of results was analyzed using an unpaired two-tailed t-test. Notably, *P < 0.05 and **P < 0.01 when compared to the control group.

The effects of varying concentrations of CRAMP-34 on 3-day-old preformed biofilms were assessed using crystal violet staining (**Figure 1A**). Concentrations of CRAMP-34 at 500 μg/mL, 250 μg/mL, and 125 μg/mL led to significant reductions in biofilm biomass by 67.23%, 72.75%, and 70.76%, respectively (*P* < 0.01). At concentrations of 62.5 μg/mL and 31.25 μg/mL, the biomass was reduced by 61.03% and 40.18%, respectively (*P* < 0.05). Notably, the reduction rate at 62.5 µg/mL aligned with our previous findings on 1-day-old preformed biofilms (Zhang et al., 2022).

The influence of different CRAMP-34 concentrations on the metabolic activity of 3-day-old preformed biofilm bacteria was determined using the thiazolyl blue tetrazolium bromide (MTT) assay (**Figure 1B**). At concentrations of 500 μg/mL, 250 μg/mL, and 125 μg/mL, there was a significant decrease in bacterial metabolic activity (*P* < 0.01) with reductions of 84.00%, 84.00%, and 76.00%, respectively. At 62.5 μg/mL, bacterial metabolic activity also declined, registering a 54.00% decrease (*P* < 0.05).

### 3.2 CLSM analysis of biofilms

**Figure 2:**
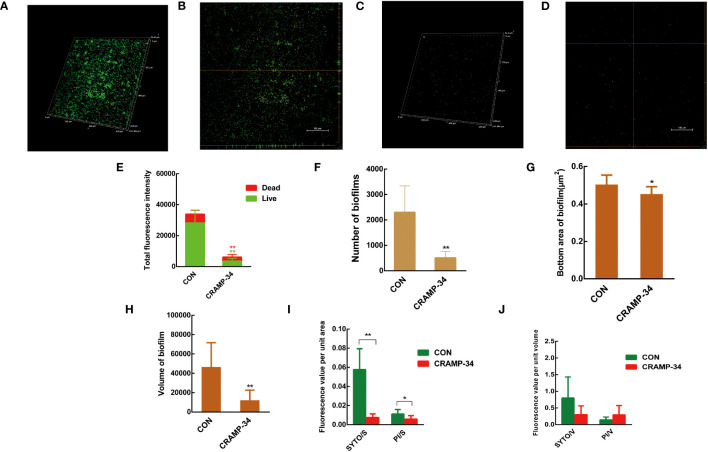
CLSM imaging of CRAMP-34 treated PAO1 prebiofilms. PAO1 biofilms were formed for 72 h at 37°C on chambered coverglass slides. biofilms were treated with CRAMP-34 for 1 h at 37°C as described and subsequently stained with SYTO 9 and PI for 20 min in the dark. **(A, B)** The 3D and orthogonal views biofilm representation in the objective of 20X in the control group. **(C, D)** The 3D and orthogonal views biofilm representation of CRAMP-34 in the objective of 20X. **(E)** The total fluorescence intensity of biofilms. **(F)** represents the number of biofilms. **(G)** The bottom area of biofilms. **(H)** represents the volume of biofilms. **(I)** The total fluorescence intensity of viable or dead bacteria per unit biofilm area. **(J)** The total fluorescence intensity of viable or dead bacteria per unit biofilm volume. Unpaired t-test (two-tailed) was used to measure statistical significance. *P < 0.05, **P < 0.01 compared with the control group.

Confocal laser scanning microscopy (CLSM) was utilized to observe preformed biofilms after a 1-hour treatment with CRAMP-34 at a concentration of 62.5 μg/mL. The biofilm related metrics were then analyzed using the Biofilm Q software. Stains used included SYTO9 (which emits green fluorescence for live bacteria) and propidium iodide (resulting in red fluorescence for dead bacteria). When compared to the control group, the biofilms in the CRAMP-34 group had notably fewer, smaller, and thinner structures (**Figures 2A–D**). An examination of the total fluorescence intensity indicated a marked reduction in both live and dead bacterial counts within the CRAMP-34 group compared to the control. Specifically, fluorescence indicating live bacteria decreased by 87.78%, while that for dead bacteria decreased by 52.50% (**Figure 2E**). The number of biofilms in the CRAMP-34 group diminished significantly, showing a 78.12% reduction compared to the control group (**Figure 2F**). Moreover, the CRAMP-34 treatment resulted in a 10.12% decrease in biofilm bottom area (**Figure 2G**), a 73.77% decline in biofilm volume (**Figure 2H**), an 85.96% reduction in the total fluorescence intensity of live bacteria per unit biofilm area, and a 45.45% drop in fluorescence for dead bacteria per unit biofilm bottom area (**Figure 2I**). However, there was no significant change in the total fluorescence intensity of either live or dead bacteria per unit biofilm volume when compared to the control (**Figure 2J**).

### 3.3 Significant differences in extracellular metabolites

**Figure 3:**
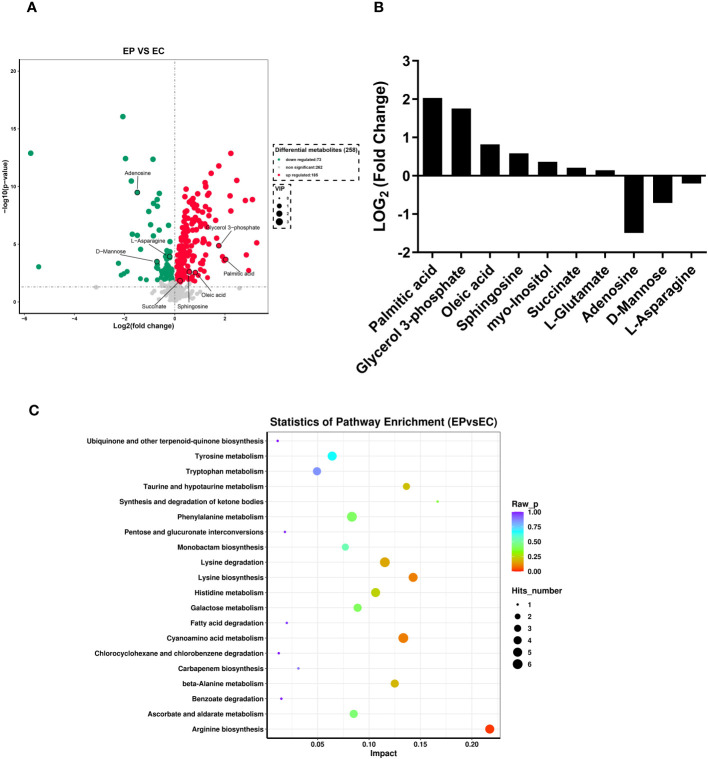
Extracellular metabolites in CRAMP-34 treated PAO1 prebiofilms. Three-day-old preformed biofilms were treated by CRAMP-34 at 62.5 μg/mL for 1 hour. There were 6 samples in both the CRAMP-34 group and the control group. The samples were analyzed by LCMS, and the data were extracted and preprocessed using Mas-terView (SCIEX). **(A)** Volcano plots showed the fold change of extracellular metabolites in CRAMP-34 group (EP) vs control group (EC). The green dots represent significantly down-regulated metabolites (73), the red dots represent significantly up-regulated metabolites (185), and grey dots represent non-significantly changed differential metabolites. **(B)** Some representative differential metabolites. **(C)** The bubble diagram was analyzed using KEGG metabolism pathway enrichment. The *p* value is the significance of enrichment of this metabolic pathway. The ordinate is the name of the metabolic pathway; the abscissa is the rich factor. The larger the rich factor, the more metabolites enriched in the pathway. The size of the dots represents the number of differential metabolites enriched into the pathway.

**Figure 4:**
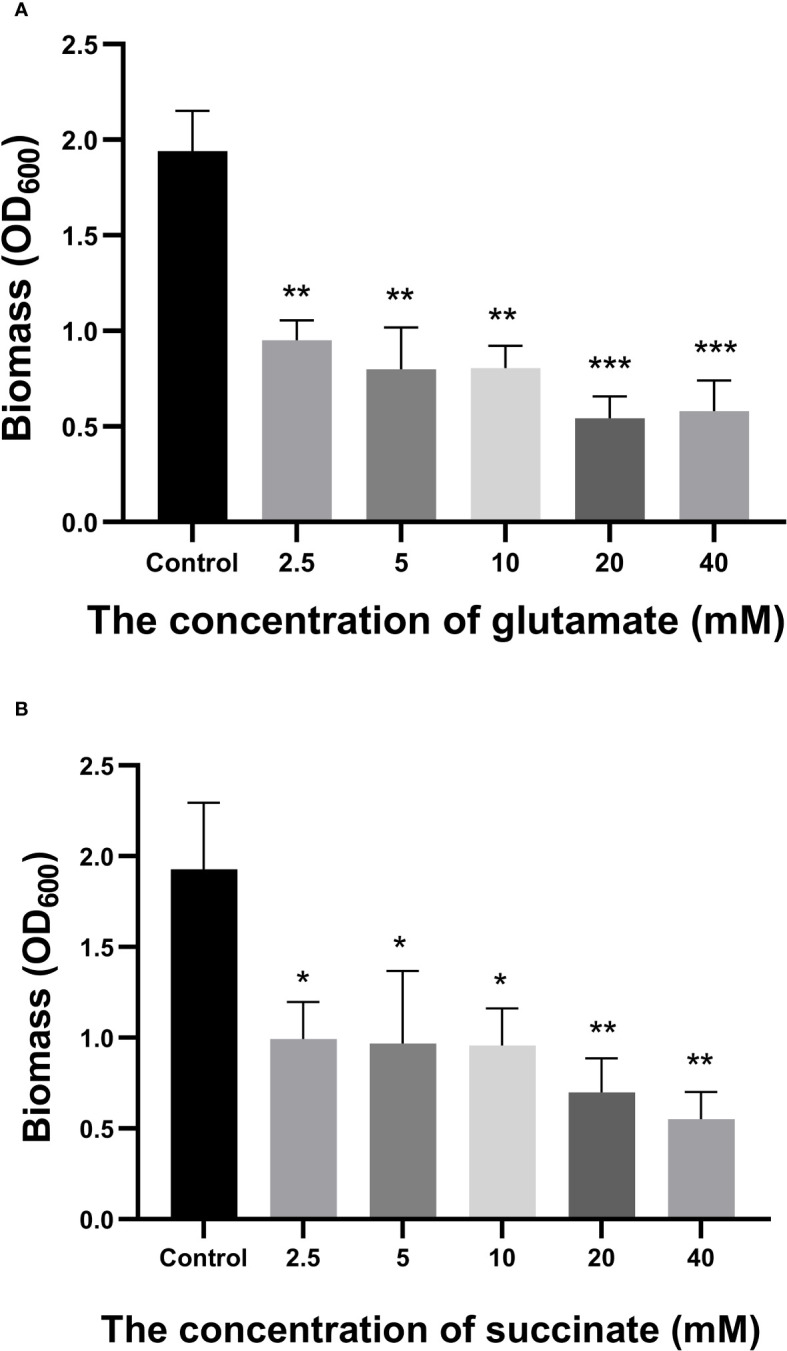
Effects of Glutamate and Succinate on PAO1 preformed biofilms. The biomass of the preformed 96-h-old biofilms treated at 37°C with different concentrations of glutamate **(A)** and succinate **(B)** were measured after 24 h. *P < 0.05 and **P < 0.01 when compared to the control group.

A total of 258 distinct extracellular metabolites exhibited significant differences when comparing the CRAMP-34 group to the Control group (EP vs EC). These differences are visualized in a volcano plot (**Figure 3A**). In this plot, green dots denote metabolites that are significantly downregulated (73 in total), red dots indicate those significantly upregulated (185 in total), while grey dots symbolize metabolites with no significant change. **Figure 3B** highlights representative differential metabolites, illustrating the Log (2) fold changes of specific substances, such as palmitic acid, glycerol 3-phosphate, and oleic acid. Differential metabolites were subjected to pathway analysis using the Kyoto Encyclopedia of Genes and Genomes database (KEGG), the top 20 metabolic pathways according to *p*-value were chosen for examination of total KEGG metabolic pathway alterations (**Figure 3C**). Metabolic enrichment analysis indicated metabolic pathways were related to amino acid biosynthesis and metabolism, including arginine biosynthesis, lysine biosynthesis, lysine degradation, taurine and hypotaurine metabolism, cyanoamino acid metabolism, beta-Alanine metabolism, etc. The primary dataset is available in the MetaboLights database. The clustering heat map and PCA score plots can be referenced in **Figures S1** and **S2** in the **Supplementary File 1**. Detailed data for all differential metabolites are provided in **Supplementary File 2**. In order to verify the extracellular metabolomics results, several significantly upregulated metabolites were selected for verification, and it was found that the exogenous addition of glutamate and succinate showed significant effects on eradicating pre-biofilms and showed a certain dose-dependent effect (validated by one way ANNOVA) (**Figure 4**).

### 3.4 CRAMP-34 combined with antibiotics acts on biofilm-dispersed bacteria

**Figure 5:**
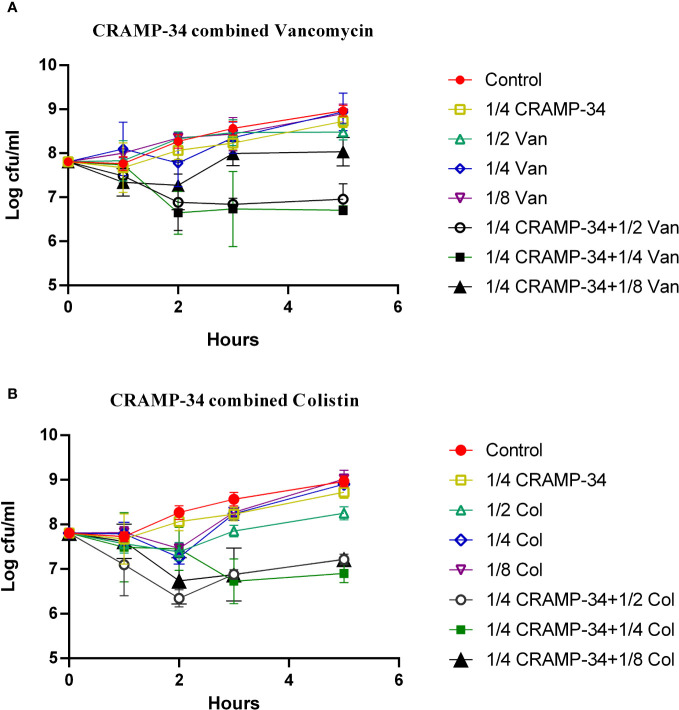
CRAMP-34 combined with antibiotics on biofilm dispersed bacteria. CRAMP-34 was used to disperse the biofilm that was pre-formed for 3 days, and the dispersed cells were collected for testing. **(A)** The time killing curve (TKC) of CRAMP-34 (1/4MIC) combined with vancomycin (1/2,1/4,1/8 MIC). **(B)** TKC of CRAMP-34 (1/4MIC) combined with colistin (1/2,1/4,1/8 MIC).

To determine if CRAMP-34 enhances the efficacy of antibiotics against bacteria dispersed from biofilms, we evaluated the combined sensitivities of 14 antibiotics with CRAMP-34 against these bacteria at subinhibitory concentrations (MIC values are provided in **Table S1** in **Supplementary File 1**). Given that dispersed bacteria transition to a planktonic state after 5 hours (Wille et al., 2020), our 5-hour bactericidal curve demonstrated that subinhibitory concentrations of CRAMP-34 (1/4MIC) in combination with colistin (1/2MIC, 1/4MIC) and vancomycin (1/2MIC, 1/4MIC) markedly reduced the dispersed bacterial count. By the established criteria, our findings indicate a synergistic effect between CRAMP-34 (1/4MIC) and both colistin (1/4MIC) and vancomycin (1/4MIC) against dispersed bacteria (**Figure 5**). Interactions with most other antibiotics were found to be additive, and those results are not detailed here.

### 3.5 CRAMP-34 delays antibiotic resistance

**Figure 6:**
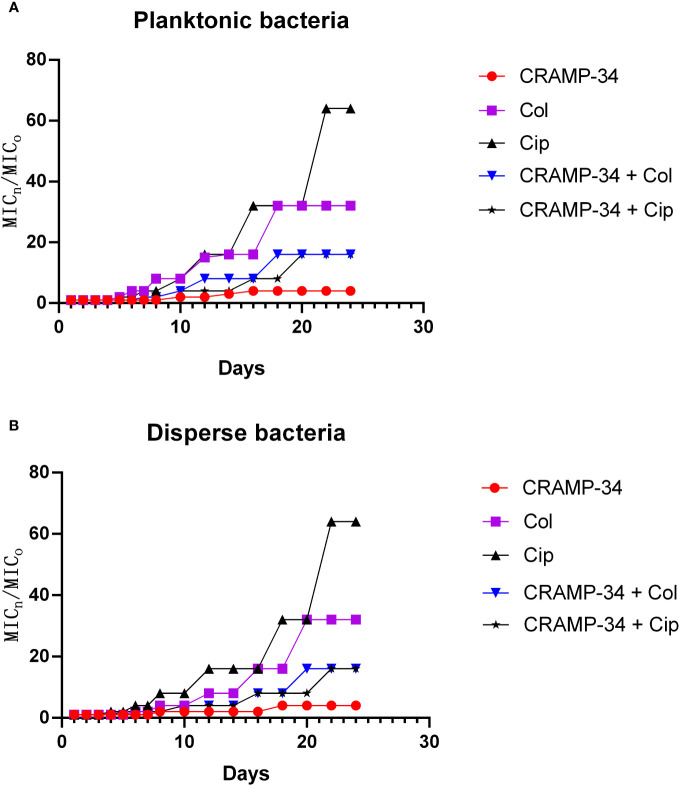
CRAMP-34 delaying antibiotic resistance in dispersed bacteria. The dispersed cells were collected for testing the ability of CRAMP-34 combined with antibiotics to delay the development of drug resistance by 25 generations of continuous induction at the sub inhibitory concentration, with planktonic cells as control. **(A)** The change multiple of the MIC value of CRAMP-34 combined with ciprofloxacin and colistin against planktonic cells. **(B)** The change multiple of the MIC value of CRAMP-34 combined with ciprofloxacin and colistin against dispersed cells.

To assess the potential of CRAMP-34 in conjunction with antibiotics to mitigate the evolution of drug resistance, we observed its effects over 25 continuous generations at subinhibitory concentrations. As depicted in **Figures 6A, B**, following 25 continuous generations with induction by CRAMP-34 at 1/4 MIC, there was only a four-fold increase in the MIC value. In contrast, a 25-generation induction with ciprofloxacin at 1/4 MIC led to a sixty-fourfold increase in the MIC value, and colistin at 1/4 MIC resulted in a thirty-twofold increase. However, when ciprofloxacin (or colistin) at 1/4 MIC was combined with CRAMP-34 at 1/4 MIC for co-induction, the MIC values were significantly reduced. These trends were consistent across both planktonic and dispersed bacteria, as illustrated in **Figures 6A, B**. In summation, CRAMP-34 appears effective in decelerating the emergence of bacterial resistance to antibiotics.

## 4 Discussion

Biofilm dispersion mechanisms can be broadly categorized into active and passive dispersion (Rumbaugh and Sauer, 2020; Wille and Coenye, 2020). Active dispersion is contingent upon a decline in intracellular c-di-GMP levels, which prompts a set of phenotypic alterations. These include the amplification of bacterial flagellar motility and diminished synthesis of EPS (Wille et al., 2020). In contrast, passive dispersion operates through stimuli that directly liberate cells from the biofilm. This encompasses the enzymatic breakdown of the biofilm matrix and external physical triggers (Wille and Coenye, 2020).

CRAMP, the sole cathelicidin identified in mice, shares structural and functional similarities with LL-37(Zhou et al., 2020). Key to its antibacterial efficacy is the amphipathic alpha-helical region stretching from Gly16 to Leu33 (Yu et al., 2002). Our research introduced an enhanced version of this molecule, termed CRAMP-34, boasting reduced cytotoxicity and augmented stability. Furthermore, our preceding investigations validated CRAMP-34’s capacity to disperse biofilms, corroborated by multi-omics analyses and a range of phenotypic observations (Zhang et al., 2022). At present, the research on antimicrobial peptides that can effectively disperse biofilms is still relatively limited. However, some studies have shown promising results with synthetic antimicrobial peptides. For instance, the antimicrobial peptide WLBU2 at 4× MIC successfully dispersed 69.7% and 81.3% of pre-formed biofilms of clinical multidrug-resistant (MDR) and standard *Pseudomonas aeruginosa* strains (ATCC 27,853), respectively (Masihzadeh et al., 2023). This study also showed that CRAMP-34 at 62.5 μg/mL (2× MIC) and 125 μg/mL (4× MIC) led to significant reductions in PAO1 biofilm biomass by 61.03% and 70.76%, respectively. To delve deeper into CRAMP-34’s anti-biofilm prowess, we deployed MTT staining to evaluate the metabolic vitality of the biofilm bacteria. Coupled with confocal laser scanning microscopy, leveraging an optimal dye ratio, presented visual insight into biofilm architecture (Zhang et al., 2022). This combined approach underlined CRAMP-34’s dose-dependent efficacy in biofilm eradication. A prior exploration into the intracellular metabonomics of biofilm bacteria spotlighted that CRAMP-34 notably reduced the expression levels of uridine diphosphate N-acetylglucosamine (UDP-GlcNAc) and UDP glucose dehydrogenase (UDP-D-G) – precursors integral to Pel and Psl synthesis. Nonetheless, while this data hints at the insufficiency in exopolysaccharide secretion, it doesn’t robustly confirm biofilm dispersion. The intricate mechanisms behind this remain elusive and warrant further investigation.

To deepen our understanding of how CRAMP-34 disperses biofilms, we probed the extracellular metabolites of biofilm bacteria post CRAMP-34 intervention. We observed a marked elevation in extracellular glutamate secretion post treatment. Prior research indicates that exogenous glutamate enhances the dephosphorylation of NicD (c-di-GMP synthetase) and amplifies the activation of BdlA and DipA proteins (both c-di-GMP degrading enzymes), culminating in reduced c-di-GMP concentrations (Basu and Sauer, 2014). A diminished c-di-GMP level is known to instigate biofilm dispersion (Rumbaugh and Sauer, 2020; Wille et al., 2020). Moreover, our metabolomics results highlighted a pronounced upsurge in extracellular succinate following CRAMP-34 application. Previous studies have highlighted that the exogenous addition of succinate and glutamate can lead to a nearly 80% reduction in surface-attached biofilm biomass, while also increasing flagella (fliC) expression in dispersed entities (Sauer et al., 2004). This aligns with our prior findings where CRAMP-34 treated PAO1 pre-biofilms demonstrated a notable drop in c-di GMP levels and increased flagella expression, thus bolstering bacterial motility (Zhang et al., 2022). We postulate that CRAMP-34 modulates glutamate and succinate metabolic levels to decrease c-di GMP concentrations and augment flagellar assembly protein expression, leading to biofilm dissolution. Additionally, while increased carbon sources were evident, carbon source depletion has also been documented to spur biofilm dispersion. For instance, glucose deprivation triggered biofilm dispersal within a mere five minutes, reaching its peak at 2 hours, and a substantial 60% of the original *P. aeruginosa* biofilm dispersed after enduring 24 hours of such deprivation (Huynh et al., 2012). Succinate is also an important intermediate product in the TCA cycle. The changed succinate indicated the disorder of TCA cycle(Zhou et al., 2019). The disordered TCA cycle will cause the insufficiency of energy supply, eventually leading to the inhibited virulence of *P. aeruginosa.* It is worth noting that there are also reports that bacteria set free from the biofilm exhibit heightened motility, virulence, and adherence compared to their planktonic counterparts (Rumbaugh and Sauer, 2020). Therefore, ineffective eradication of these dispersed bacteria could escalate the risk of infection spread, especially when employing dispersants to combat biofilm-associated infections.

This study unveiled a pronounced surge in the expression of extracellular metabolites post-CRAMP-34 treatment, specifically myoinositol, palmitic acid, and oleic acid. Notably, exogenous myo-inositol has been associated with amplified sensitivity of *Aeromonas hydrophila* to florfenicol (Zhao et al., 2018). Further studies have corroborated that the extracellular levels of palmitic and oleic acids in antibiotic-sensitive strains considerably surpass those in multi-drug resistant counterparts (Kocak et al., 2022). In addition, glutamate is a crucial component in the synthesis of glutathione. Variations in glutamate levels may lead to the disorder of glutathione, subsequently enhancing oxidative stress (Zhou et al., 2023). The increase in glutamate levels after the intervention of CRAMP-34 also indicates that the heightened oxidative stress has the potential to disrupt the structure of cell membranes, alter membrane permeability, and ultimately enhance the sensitivity to conventional antibiotics. These observations hint at the potential of CRAMP-34 to heighten the antibiotic sensitivity of biofilm-dispersed bacteria. In alignment with this, our drug sensitivity assays illustrated that the combined application of subinhibitory concentrations of CRAMP-34 with antibiotics, namely colistin and vancomycin, achieved a significant decrease in dispersed bacteria counts. Our prior research had also discerned a noteworthy reduction in PAO1 biofilm formation when CRAMP was paired with colistin (Zhang et al., 2021). Furthermore, the synergistic antimicrobial action of LL-37/CAMA and ciprofloxacin against *P. aeruginosa* biofilm bacteria has been documented (Dosler and Karaaslan, 2014), although comprehensive studies regarding the combined antibiotic sensitivity toward dispersed bacteria remain scarce. Collectively, our findings underscore the promise of CRAMP-34 as a potent biofilm dispersant, which, when amalgamated with antibiotics, holds great potential for ad-dressing biofilm-associated infections.

It is important to note that this present study also found significant increased glycerol 3-phosphate and sphingosine in the extracellular metabolites after CRAMP-34 treatment. Glycerol 3-phosphate is the key precursor to build membrane lipids, and sphingosine is involved in the synthesis of sphingolipids which is a class of cell membrane lipids (Kocak et al., 2022). It is speculated that the CRAMP-34 may cause the destruction of bacterial cell membrane, thereby stimulating the massive synthesis of new bacterial cell membranes. Moreover, this study showed that CRAMP-34 combined with antibiotics inhibits dispersed bacteria but whether the prolonged exposure to sub-inhibitory concentration of CRAMP-34 leads to the increased resistance of dispersed bacteria is still unknown. Therefore, to investigate drug resistance, we continuously exposed PAO1 to CRAMP-34 at sub-inhibitory concentrations across 25 generations Notably, we did not observe obvious drug resistance of CRAMP-34, and sub-MIC CRAMP-34 delayed the production of antibiotic resistance (ciprofloxacin and colistin), which was observed in both planktonic bacteria and dispersed bacteria.

## 5 Conclusion

Taken together with prior research, our findings indicate that CRAMP-34 facilitates the transition of PAO1 bacteria from a biofilm configuration to a planktonic one. It is hypothesized that this is driven by an up-regulation of extracellular glutamate and succinate metabolism, mediated via diminished c-di-GMP levels, culminating in biofilm dispersal. Moreover, heightened levels of myoinositol, palmitic acid, and oleic acid in extracellular metabolites amplify the antibiotic sensitivity of dispersed bacteria, particularly toward colistin and vancomycin. This effect also extends to delaying bacterial resistance to both colistin and ciprofloxacin. Given these insights, there is potential in advancing CRAMP-34 as a biofilm dispersant in tandem with antibiotics. This offers a novel therapeutic strategy for preempting and treating infections linked with biofilms.

## Data availability statement

The datasets presented in this study can be found in online repositories. The names of the repository/repositories and accession number(s) can be found in the article/**Supplementary Material**.

## Author contributions

SW: Data curation, Methodology, Visualization, Writing – original draft. CM: Data curation, Methodology, Validation, Visualization, Writing – review & editing. JL: Methodology, Validation, Visualization, Writing – review & editing. PC: Data curation, Methodology, Visualization, Writing – review & editing. YZ: Methodology, Validation, Visualization, Writing – review & editing. LP: Funding acquisition, Supervision, Writing – review & editing. LF: Funding acquisition, Supervision, Writing – review & editing. YY: Funding acquisition, Project administration, Writing – review & editing. DX: Methodology, Validation, Writing – review & editing. SZ: Resources, Supervision, Writing – review & editing. JQ: Resources, Supervision, Writing – review & editing. YH: Methodology, Visualization, Writing – review & editing. HY: Investigation, Methodology, Validation, Visualization, Writing – review & editing, Supervision. HC: Conceptualization, Funding acquisition, Methodology, Project administration, Supervision, Writing – review & editing, Resources.
