# Supplementary material for: Impact of CRAMP-34 on Pseudomonas aeruginosa biofilms and extracellular metabolites
Source: Front Cell Infect Microbiol. 2023 Dec 13;13:1295311. doi: 10.3389/fcimb.2023.1295311 (PMC10757720; doi:10.3389/fcimb.2023.1295311)
Supplement: Supplementary file 1 [file DataSheet_1.docx]

Supplementary Material

# Supplementary Figures and Tables

## Supplementary Figures

**
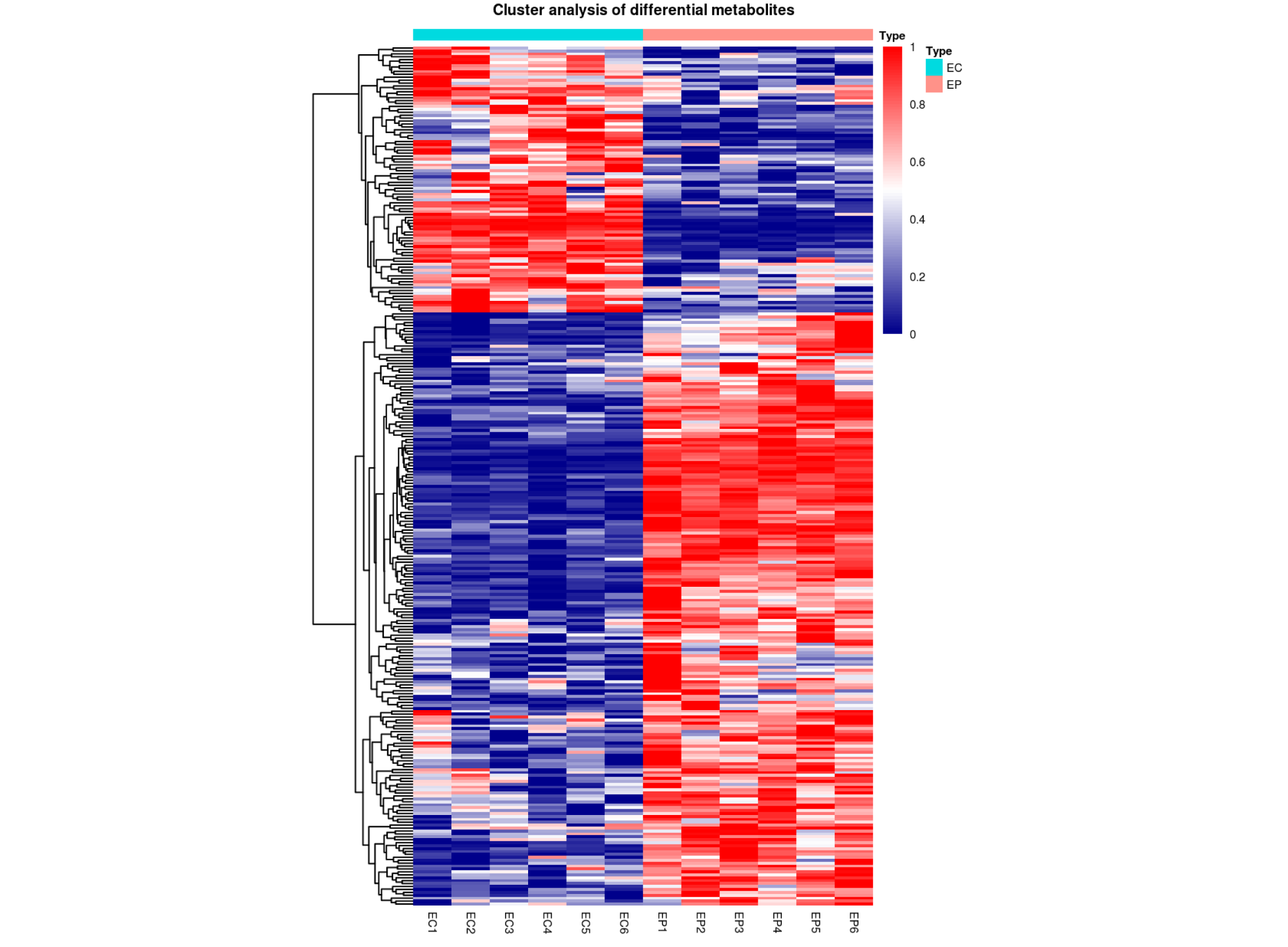
**

**Supplementary Figure 1.** **The clustering heat map of extracellular metabolites** **from metabolomics.** The 3-day PAO1 pre-biofilms was treated with CRAMP-34 (62.5 μg/mL) for 1h, and the untreated biofilms was used as the control. All experiments were performed at least in triplicate, and there were 6 samples in both the CRAMP-34 group (EP) and the control group (EC).

**
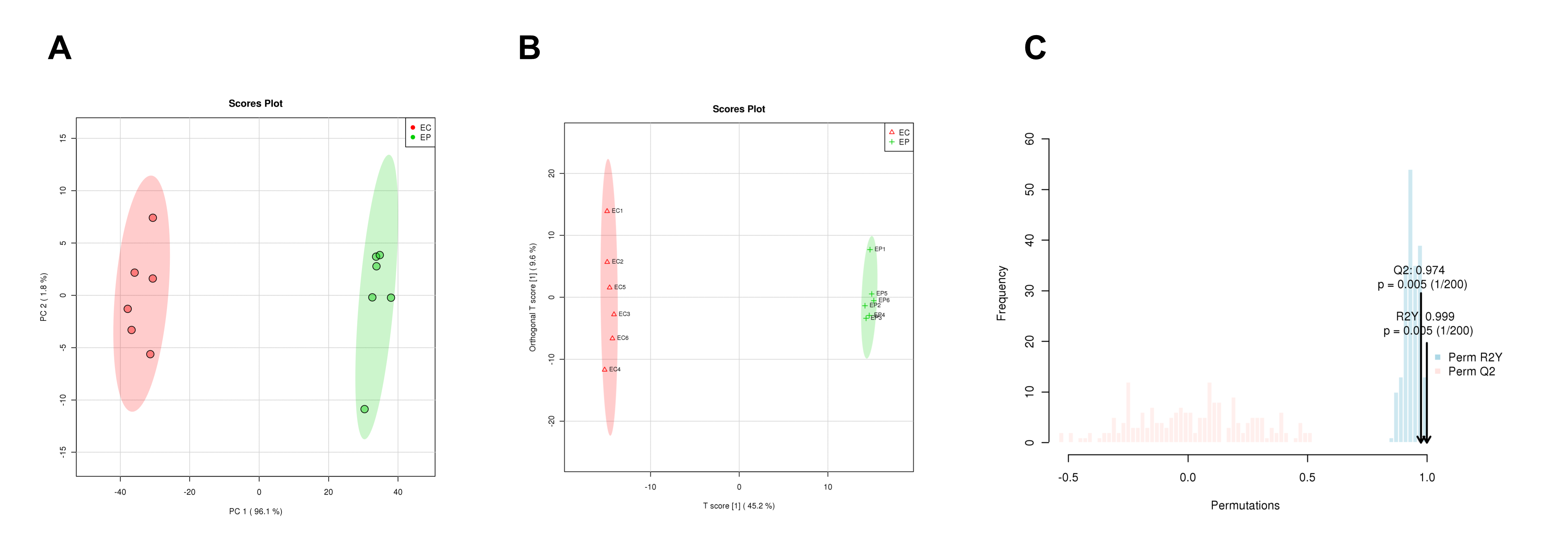
**

**Supplementary Figure 2.** **Principal component analysis (PCA) and** **orthogonal partial least square-discriminate analysis (****OPLS-DA) from metabolomics.**The 3-day PAO1 pre-biofilms was treated with CRAMP-34 (62.5 μg/mL) for 1h, and the untreated biofilms was used as the control. All experiments were performed at least in triplicate, and there were 6 samples in both the CRAMP-34 group (EP) and the control group (EC). (A) The score scatter plot of principal component analysis. (B) Orthogonal partial least square-discriminate analysis. (C) Validation of orthogonal partial least square-discriminate analysis models.

## Supplementary Tables

**Table S1** **Minimum inhibitory concentrations of various drugs against PAO1**

| Drug | MIC(μg/mL) |
| --- | --- |
| CRAMP-34 | 31.25 |
| Meropenem | 0.5 |
| Imipenem | 2 |
| Piperacillin | 2 |
| Ceftazidime | 1 |
| Cefoquinol | 2 |
| Cefoperazone | 4 |
| Gentamicin | 0.5 |
| Amikacin | 0.25 |
| Azithromycin | 16 |
| Roxithromycin | 512 |
| Ciprofloxacin | 0.031 |
| Enrofloxacin | 0.5 |
| Colistin | 0.25 |
| Vancomycin | 1024 |
